# LPS Responsiveness and Neutrophil Chemotaxis In Vivo Require PMN MMP-8 Activity

**DOI:** 10.1371/journal.pone.0000312

**Published:** 2007-03-21

**Authors:** Angus M. Tester, Jennifer H. Cox, Andrea R. Connor, Amanda E. Starr, Richard A. Dean, Xose S. Puente, Carlos López-Otín, Christopher M. Overall

**Affiliations:** 1 University of British Columbia Centre for Blood Research, Departments of Oral Biological and Medical Sciences, and Biochemistry and Molecular Biology, University of British Columbia, Vancouver, British Columbia, Canada; 2 Department Bioquimica y Biologia Molecular, Instituto Universitario de Oncologia, Universidad de Oviedo, Oviedo, Spain; New York University School of Medicine, United States of America

## Abstract

We identify matrix metalloproteinase (MMP)-8, the polymorphonuclear (PMN) leukocyte collagenase, as a critical mediator initiating lipopolysaccharide (LPS)-responsiveness *in vivo*. PMN infiltration towards LPS is abrogated in *Mmp8*-null mice. MMP-8 cleaves LPS-induced CXC chemokine (LIX) at Ser^4^∼Val^5^ and Lys^79^∼Arg^80^. LIX bioactivity is increased upon N-terminal cleavage, enhancing intracellular calcium mobilization and chemotaxis upon binding its cognate receptor, CXCR2. As there is no difference in PMN chemotaxis in *Mmp8*-null mice compared with wild-type mice towards synthetic analogues of MMP-8-cleaved LIX, MMP-8 is not essential for extravasation or cell migration in collagenous matrices *in vivo*. However, with biochemical redundancy between MMPs 1, 2, 9, and 13, which also cleave LIX at position 4∼5, it was surprising to observe such a markedly reduced PMN infiltration towards LPS and LIX in *Mmp8*-/- mice. This lack of physiological redundancy *in vivo* identifies MMP-8 as a key mediator in the regulation of innate immunity. Comparable results were found with CXCL8/IL-8 and CXCL5/ENA-78, the human orthologues of LIX. MMP-8 cleaves CXCL8 at Arg^5^-Ser^6^ and at Val^7^-Leu^8^ in CXCL5 to activate respective chemokines. Hence, rather than collagen, these PMN chemoattractants are important MMP-8 substrates *in vivo*; PMN-derived MMP-8 cleaves and activates LIX to execute an *in cis* PMN-controlled feed-forward mechanism to orchestrate the initial inflammatory response and promote LPS responsiveness in tissue.

## Introduction

Polymorphonuclear neutrophils (PMNs) are crucial inflammatory leukocytes in host protection from infection, where their primary role is in phagocytosis and killing of bacteria, fungi and protozoa, and in wound debridement and healing [1], [2]. Given these critical roles of PMNs, it has long been recognised that neutropenic patients are at greater risk of infection [3], and that is often observed after intensive cancer chemotherapy [4], [5]. Proteolysis of phagosome contents and damaged extracellular matrix are key PMN actions in inflammation. Cell migration, crossing basement membrane and connective tissue matrix barriers are other aspects of PMN function traditionally thought to require proteolytic activity [6]. Additionally, PMNs are a source of chemotactic factors that guide the recruitment of specific and non-specific immune effector cells [7] and so these first line defence cells play key roles in innate and acquired immunity.

Of the two major chemokine subfamilies that provide directional cues for leukocyte migration and activation [8], the CXC chemokines predominantly influence PMNs and T-lymphocytes whereas the CC chemokines are active on monocytes, basophils and eosinophils [9]. The expression of CXC chemokines is rapidly upregulated during acute inflammatory responses, such as that initiated by the endotoxin lipopolysaccaride (LPS) [10]–[13]. A subset of the CXC chemokines are characterised by an ELR (glutamic acid-leucine-arginine) sequence proximal to the conserved CXC motif. ELR is essential for binding CXC-receptors (CXCR) 1 and 2 [14] leading to PMN activation, degranulation and release of proteases [15]. The murine ELR^+^ CXC chemokines act through a single receptor that is homologous to human CXCR2 [16]. In humans there are seven ELR^+^ CXC chemokines; CXCL8/interleukin-8 (IL-8); CXCL7/neutrophil-activating peptide-2 (NAP-2); CXCL6/granulocyte chemotactic protein-2 (GCP-2); CXCL5/epithelial cell-derived neutrophil activating peptide-78 (ENA-78); and CXCL1, -2 and -3 (also known as growth-related oncogenes (GRO) α, -β, and -γ). Only CXCL8/IL-8, the most potent of these chemokines, and CXCL6/GCP-2 bind CXCR1, whereas all members signal through the closely related receptor CXCR2 [14]. Mice lack a homologue of CXCL8/IL-8 having only four ELR^+^ CXC chemokines: LPS-induced CXC chemokine (LIX), the most abundant and potent of the murine chemokines and regarded as the orthologue of CXCL8 [17]; keratinocyte-derived chemokine (KC); macrophage inflammatory protein-2 (MIP-2); and dendritic cell inflammatory protein-1 (DCIP-1). Physiological N-terminal cleavage of chemokines modifies their bioactivity—either enhancing activity of the ELR^+^ CXC chemokines [15] or generating potent receptor antagonists from the CC chemokines CCL2, -7, -8 and -13 (also known as macrophage chemotactic proteins 1 to 4) [18], [19]. Although several candidate proteases are proposed for ELR^+^ CXCL proteolytic activation, none have been validated *in vivo*.

Matrix metalloproteinases (MMPs) are traditionally associated with extracellular matrix protein degradation in many physiological and pathological processes, including inflammation, bacterial infection, wound healing, and cancer cell invasiveness (reviewed in ref [20]). However, it is now clear that MMPs mediate homeostasis of the extracellular environment [21] by modulating the biological activity of many bioactive molecules involved in cell function [22], [23], innate immunity [24] including chemokines [18], [19], [23], [25]–[27], TNF-α [22], [28], α-defensin [29], and mannose binding lectin [30], and in tumour initiation and progression. Inflammation in cancer, particularly macrophage infiltration and MMP-9 release, generates a microenvironment advantageous to neoplastic progression [31], with recent evidence indicating that a PMN source of MMP-9 can also promote tumorigenesis [32].

MMP-8 (human: P22894, mouse: CAA73786, rat: AJ007288), the neutrophil collagenase, is produced primarily by PMNs and is released from the specific granules at sites of inflammation [33]. *Mmp8*-null mice have no overt phenotype, with normal embryonic development, fertility, and long-term survival [34]. In contrast to other MMP deficient mice [35], *Mmp8*-null mice challenged with carcinogens showed a markedly increased susceptibility to tumorigenesis [34], but this only occurred in male mice. This was the first report of a MMP having a protective role in tumorigenesis, so validating MMP-8 as an anti-target in cancer therapy [35].

As a potent type I collagenase [36], [37] it had been predicted that mice lacking the *Mmp8* gene would show reduced PMN migration through collagenous matrices [34]. Indeed, at the tumor stromal interface an abnormal inflammatory response is observed, characterised by an initially delayed and then a more diffuse PMN influx in the *Mmp8*-null mice [34]. However, once established there was a prolonged chronic accumulation of PMNs that did not dissipate. Overall, this phenotype is reversed following transplantation of wild type bone marrow, confirming that the absence of MMP-8 produced by PMNs and not a tissue or tumour source resulted in the higher incidence of tumors. In a model of TNF-induced acute hepatitis, MMP-8 deficient mice showed dampened levels of PMN infiltration into the liver that was postulated to result from reduced LIX mobilization from an unidentified binding protein in the matrix [38]. Together, these studies suggest a coordinating role for MMP-8 in physiological leukocyte trafficking both in acute and chronic inflammation, either through cleavage of collagen or chemokine binding proteins [38], or by processing of bioactive molecules, such as LIX [34] to control PMN migration or longevity.

Here we have investigated the role of MMP-8 in PMN recruitment during acute inflammation using LPS responsiveness as a trigger; the PMN influx was abrogated in the *Mmp8*-/- mouse. LIX is identified as a key inflammatory substrate of MMP-8 where N-terminal processing by MMP-8 activates the chemokine and so increases PMN chemotaxis and LPS responsiveness *in vitro* and *in vivo*. Although these activities are reduced in the *Mmp8*-null mouse, the loss of neutrophil collagenolytic activity did not alter *in vitro* PMN chemokinesis or *in vivo* chemotaxis when challenged with truncated LIX or truncated CXCL8/IL-8 chemokines. Hence, these data reveal a new auto-regulatory mechanism of PMN chemotaxis that is initiated by MMP-8 release from PMNs and executed, directly or indirectly, by the proteolytic activation of LIX in mice and CXCL8 and CXCL5/ENA-78 in man. This drives further PMN migration in a novel feed-forward mechanism that, remarkably, is a major determinant of LPS responsiveness.

## Results

### LPS induced PMN response in mice

**Figure 1:**
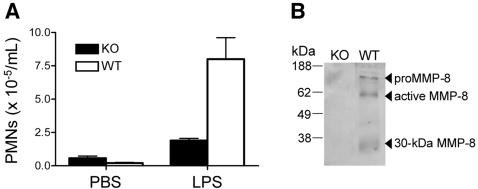
Impaired PMN responsiveness to LPS in MMP-8 deficient mice. (A) Infiltration of PMNs *in vivo* in response 1 µg of LPS (n = 8) or phosphate buffered saline control (n = 4) injected into the air pouch of male *Mmp8*-/- (black bar) and wild type mice (white bar) was assessed 8 h post-injection. The PMN influx was quantitated by myeloperoxidase activity. Error bars, standard error. (B) Western blot analysis of murine MMP-8 in LPS-treated air pouch PMN lysates corresponding to 50,000 cells per lane.

To ascertain the role of MMP-8 in PMN cell migration and LPS responsiveness *in vivo* we compared *Mmp8*-/- with *Mmp8*+/+ mice. The PMN influx was significantly reduced (*P*≤0.005) when LPS was injected in air pouches formed under the dorsal skin of the MMP-8 knockout mice compared with wild type mice (Figure 1A). This was observed in both male and female mice, although the PMN infiltrate was generally greater in the females towards both LPS and the PBS control. Hence, the reduced PMN migration and accumulation in *Mmp8*-/- mice reveals a critical role for this PMN-specific protease in neutrophil function in acute inflammation. Notably, MMP-8 was only detected in cell lysates from LPS-treated air pouches of wild type mice, revealing both the pro and active forms of the enzyme at 85 and 65 kDa, respectively (Figure 1B). A 30-kDa inactive degradation product of MMP-8 was also detected, as observed previously [39].

### MMP-8 processing of murine ELR^+^ CXC chemokines

**Figure 2:**
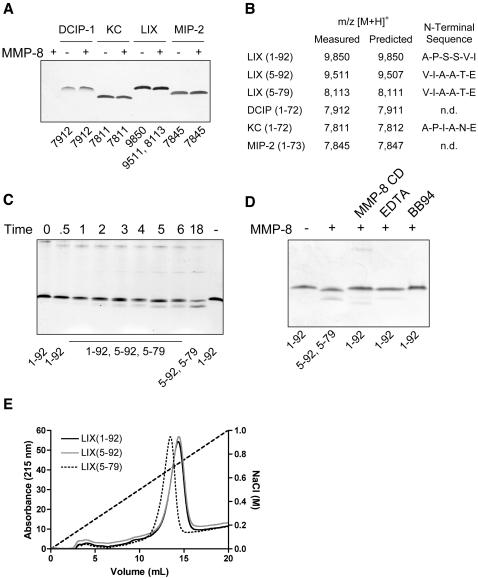
MMP-8 Cleavage of LIX. (A) Tris-tricine 15% SDS-PAGE gel analysis of MMP-8 cleavage of murine CXCR2-binding chemokines DCIP-1, KC, LIX and MIP-2. The m/z [M+H]^+^ of reaction products are as shown. (B) Identification of cleavage products by MALDI-TOF mass spectrometry and NH_2_-terminal Edman sequencing. *n.d.*, not determined. (C) Tris-tricine 15% SDS-PAGE and MALDI-TOF analysis of LIX cleavage products generated over time (h) by MMP-8. (D) Tris-tricine 15% SDS-PAGE and MALDI-TOF analysis of LIX cleavage by MMP-8 in the presence of 10-fold molar excess of recombinant hemopexin C-domain (*MMP-8 CD*), 10 µM EDTA, or 10 µM BB94. (E) Heparin Sepharose AKTA chromatograms of synthetic analogues of MMP-8 cleavage products of LIX eluted with a linear gradient of NaCl as indicated.

We investigated the potential role of MMP-8 to modulate the activity of the cognate ELR^+^ ligands of CXCR2 that may underlie the defect in LPS-induced PMN migration. The four murine ELR^+^ CXC chemokines LIX, KC, MIP-2, and DCIP-1 were incubated with recombinant MMP-8. Of these, LIX was the only chemokine susceptible to proteolytic processing (Figure 2A)—KC, MIP-2, and DCIP-1 were MMP-8-resistant even at high enzyme:substrate ratios (1∶10) and after prolonged incubation times revealing protease substrate specificity. MALDI-TOF mass spectrometry analysis of the LIX cleavage products showed that MMP-8 processed the chemokine at two sites. The major product of 9,511 Da represents a deletion of the first four NH_2_-terminal amino acid residues. Edman sequencing confirmed the deconvoluted MALDI-TOF data (Figure 2B). Hence, MMP-8 cleaves the 92-amino acid residue LIX between Ser∼Val at position 4-5 to generate a new NH_2_-terminus at Val^5^ that we designate LIX (5-92). With a measured mass of 8,113 Da, the second cleavage product was processed near the COOH-terminus after Lys^79^, resulting in the removal of 13 amino acid residues and generating the truncated form of LIX designated LIX (5-79) (Figure 2B). LIX (1-79) was never detected. Edman sequencing confirmed the deconvoluted mass spectrometry analysis of the Ser^4^∼Val^5^ amino-terminal cleavage site of LIX (5-79).

As determined by MALDI-TOF mass spectrometry, the NH_2_- and COOH-terminal processing of LIX was efficient; the cleavage products were first detected by mass spectrometry after 1 h with no full-length chemokine remaining at 18 h (Figure 2C). The COOH-terminal cleavage product was not detected in the absence of NH_2_-terminal processing at any time point indicating it occurs subsequent to NH_2_-terminal cleavage. Although the C-terminal cleavage was at an unusual highly cationic site ^75^KKKAK∼RNALA^84^ it was confirmed to be MMP dependent as two MMP inhibitors, EDTA and the synthetic hydroxamate small molecule chemical inhibitor BB94, blocked all LIX processing with no cleavage products detected by MALDI-TOF mass spectrometry or Tris-tricine SDS-PAGE (Figure 2D). Chemokine cleavage is often enhanced by MMP exosite interactions [40]. For instance, the hemopexin C domain of MMP-2 greatly increases the catalytic rate constants of CCL2, -7, -8 and -13 cleavage [19]. When molar excess of recombinant MMP-8 hemopexin C-domain was added in the cleavage assays, we likewise found that all LIX processing by MMP-8 was abrogated revealing that a binding site for LIX was present on the hemopexin C domain of MMP-8 (Figure 2D). The C-terminal cleavage at Lys^79^∼Arg^80^ only slightly reduced heparin affinity (Figure 2E) consistent with the removal of just two basic residues in the cleaved peptide ^80^ **R**NALAVE**R**TASVQ^92^ while the N-terminal truncation had no effect on heparin binding.

### Processing of LIX by other MMPs

**Figure 3:**
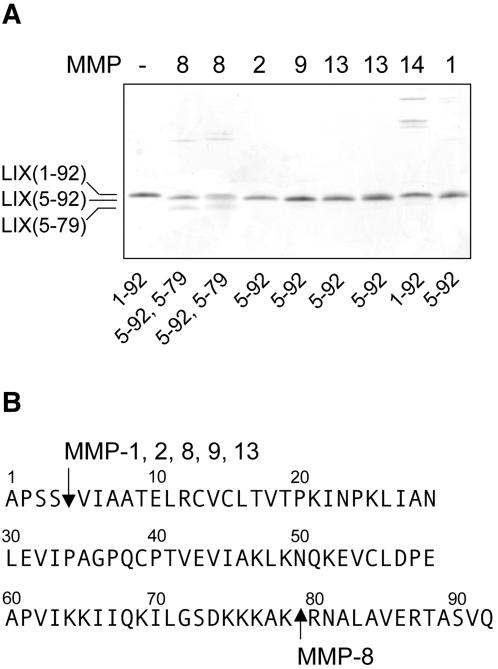
LIX is selectively cleaved by multiple MMPs. (A) Tris-tricine SDS-PAGE and MALDI-TOF mass spectrometry analysis of MMP processing of LIX (enzyme:substrate ratio of 1∶100 (w:w)) showing that MMPs 1, 2, 8, 9 and 13 cleave LIX at position 4–5, whereas MMP-14 does not, and that only MMP-8 also cleaves at position 79∼80. Cleavage assays of rodent MMP-8 and MMP-13 are shown in the second *8* and *13* lanes. (B) Cleavage data are summarised using the full-length sequence of LIX.

Proteolytic screening of LIX cleavage by several MMPs showed that MMP-9, a prominent PMN MMP, processed LIX at Ser^4^∼Val^5^ (Figure 3). In addition to MMP-9, other important stromal, endothelial and leukocytic MMPs 1, -2, and -13 could also process LIX at Ser^4^∼Val^5^ (Figure 3), but like MMP-8, did not cleave KC, MIP-2, or DCIP-1 (not shown). Similar redundancy has been shown with MMPs for CXCL12 (also know as SDF-1) [25] and for CCL2, -7, -8 and -13 [19]. Protease selectivity was also shown with MMP-14, the RNA of which is also expressed by PMNs (data not shown), but was incapable of processing LIX at any position (Figure 3A). Further, only MMP-8 could process the C-terminus of LIX at Lys^79^∼Arg^80^.

### Effect of LIX processing on *in vitro* biological activity

**Figure 4:**
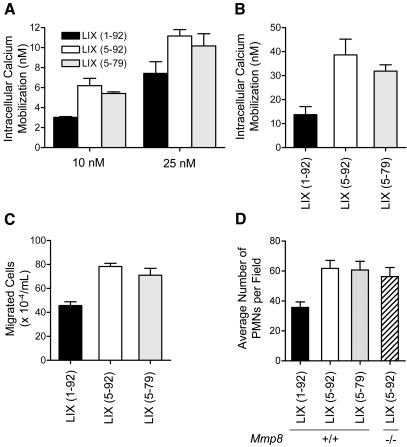
*In vitro* cellular responses to MMP-8 cleaved LIX. (A) Enhanced intracellular calcium mobilization was induced by LIX (5-92) and LIX (5-79) compared to full-length LIX (1-92) in recombinant CXCR2-expressing B300-19 cells and (B) PMNs isolated from *Mmp8*-/- mice (100 nM chemokine). (C) By transwell cell migration assay, both LIX (5-92) and LIX (5-79) truncated forms are more potent chemoattractants compared with the full-length LIX (1-92) for both CXCR2-expressing B300-19 cell transfectants and (D) murine PMNs isolated from either *Mmp8*+/+ or *Mmp8*-/- mice, all at 10 nM chemokine concentration.

Upon binding to the receptor CXCR2, LIX mobilizes intracellular Ca^++^ ion stores. As measured in recombinant CXCR2-expressing murine pre-B 300-19 cells, synthetic analogues of the MMP-truncated forms of LIX (5-92) and LIX (5-79) both induced an ∼2-fold greater intracellular Ca^++^ ion release compared to full-length LIX (1-92) (Figure 4A). This was confirmed using murine *Mmp8*-/- PMNs where an even greater stimulation in Ca^++^ ion release was observed upon binding LIX (5-92) compared with full-length LIX (Figure 4B). Functionally, this translated into enhanced chemoattraction for the CXCR2-expressing pre-B 300-19 cells by both of the truncated forms of LIX versus the full-length chemokine (Figure 4C), and in purified PMNs from *Mmp8*+/+ mice compared to the unprocessed LIX (1-92) (Figure 4D). Notably, PMNs isolated from *Mmp8*-/- mice migrated towards LIX (5-92) in a comparable manner to the *Mmp8*+/+ PMNs (Figure 4D and data not shown) indicating that the locomotor functions of PMNs isolated from both wild type and MMP-8 knock out mice were equivalent *in vitro* and unaffected by the presence or absence of MMP-8.

### *In vivo* PMN chemotaxis towards LIX

**Figure 5:**
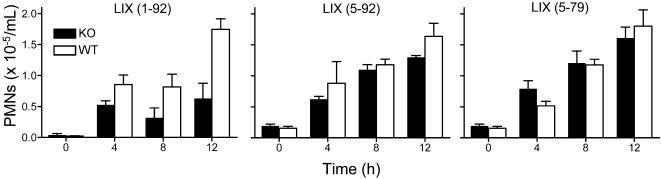
MMP-8 is required for PMN chemotaxis towards LIX *in vivo*, but is not required for PMN cell migration. PMN infiltration was greatly reduced in response to full-length LIX (1-92) injected into the dorsal skin air pouch of *Mmp8*-/- mice (black bars) compared to wild type mice (white bars). PMN numbers were calculated from myeloperoxidase assay after sacrifice at 0, 4, 8 and 12 h following injection of chemokine (n = 4). Unaltered PMN cell migration into air pouches of *Mmp8*-/- mice (black bars) compared to wild type mice (white bars) injected with MMP-cleaved analogues of LIX (5-92 or 5-79) reveals no intrinsic cell kinesis defects or chemotactic ability in the PMNs of the knock out mice.

Infiltration of PMNs towards LIX (1-92) injected in a dorsal skin air pouch of *Mmp8*-/- mice was impaired at all time points compared to PMN infiltration in wild type mice, with an ∼2-fold lower number of PMNs seen at 8 and 12 h in knock out compared with wild type mice (Figure 5). In contrast, when LIX (5-92) or LIX (5-79) were used as chemoattractants there was no significant difference in PMN infiltration into the air pouches of wild type and mice lacking MMP-8 (Figure 5). This indicates that MMP-8 activity is not essential for blood vessel extravasation and PMN cell migration *in vivo* and that there is little physiological redundancy by PMN MMP-9, or from tissue MMPs that we found competent in cleaving and activating LIX in the biochemical context *in vitro*.

### MMP-8 processes and activates IL-8 and ENA-78

**Figure 6:**
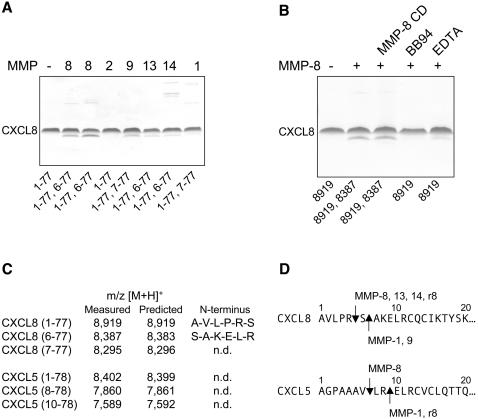
MMP processing of CXCL8 and CXCL5. (A) Tris-tricine 15% SDS-PAGE gel and MALDI-TOF mass spectrometry analysis of CXCL8 cleavage products after assay with the indicated human MMPs at an enzyme to substrate ratio of 1∶100. Cleavage assays of rodent MMP-8 are shown in the second *8* lane. The NH_2_-terminus of the CXCL8 truncated forms were deconvoluted from the mass spectrometry data and confirmed by Edman sequencing (as shown in C) with the CXCL8 forms identified shown below the corresponding gel lanes. *1-77*, full-length CXCL8. (B) The effect of recombinant MMP-8 hemopexin C domain (*MMP-8 CD*), 10 µM EDTA, or 10 µM BB94 on CXCL8 cleavage by human MMP-8 as determined by Tris-tricine 15% SDS-PAGE and MALDI-TOF mass spectrometry. The m/z [M+H]^+^ of reaction products is as shown. (C) Identification of the NH_2_-termini of MMP cleavage products of CXCL8 and CXCL5 by MALDI-TOF mass spectrometry and NH_2_-terminal Edman sequencing. *n.d.*, not determined. (D) Location of the various MMP cleavage sites on the CXCL8 and CXCL5 sequences.

Our experimental data suggest that upon LPS-induced release of LIX and resultant PMN chemoattraction a feed-forward PMN activation mechanism operates *in vivo*. MMP-8 released from degranulating PMNs at the site of challenge fully activates LIX in the tissue to further enhance PMN migration towards the LPS stimulus. To ascertain whether a similar autologous CXCR2 ligand activation mechanism occurs in man we assessed every human CXCR2 ligand for MMP-8 cleavage. Of the seven ELR^+^ CXC chemokines (CXCL8/IL-8, CXCL7/NAP-2, CXCL6/GCP-2, CXCL5/ENA-78, CXCL3/GROγ, CXCL2/GROβ and CXCL1/GROα) only CXCL8 and CXCL5 were processed by MMP-8. By MALDI-TOF MS and confirmation by Edman sequencing, CXCL8 was NH_2_-terminally processed by MMP-8 at Arg^5^∼Ser^6^ to generate CXCL8 (6-77) (Figure 6A, C) whereas CXCL5 was cleaved at Val^7^∼Leu^8^ to generate CXCL5 (8-78) (Figure 6C). There were no COOH-terminal cleavages detected. Another difference from the murine system was that CXCL8 cleavage was not hemopexin C domain dependent—proteolysis was not inhibited in the presence of a molar excess of hemopexin C domain (Figure 6B).

**Figure 7:**
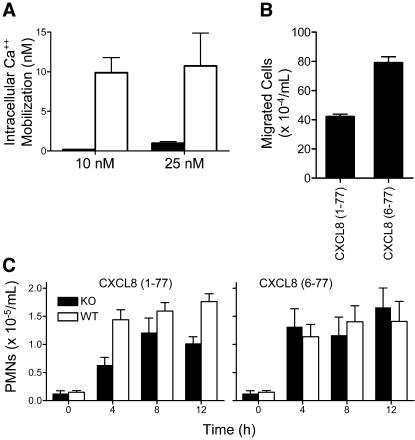
Enhanced bioactivity of MMP-truncated CXCL8 *in vitro* and *in vivo.* (A) Ca^++^ ion mobilization with 10 nM CXCL8 and (B) chemotaxis of CXCR2-transfected B300-19 cells stimulated with 10 nM full-length CXCL8 (1-77) and CXCL8 (6-77) synthetic analogue of MMP-8-cleaved CXCL8. (C) Time course of air pouch PMN influx in response to CXCL8 (1-77) and CXCL8 (6-77) in *Mmp8*-/- mice (black) and wild type (white) mice as quantitated by myeloperoxidase assay.

To determine protease specificity, other MMPs were screened for cleavage of CXCL5 and CXCL8. Only MMP-1 and MMP-8 cleaved CXCL5, with MMPs 1, 8, 9, 13 and 14 cleaving CXCL8. Some differences in cleavage site specificity were identified, but all cleavages were NH_2_-terminal to the ELR motif (Figure 6D) and none cut in the C-terminal α-helix, as occurs for LIX. Consistent with previous studies [15], [26], [27] and our results with MMP-8 cleavage of LIX, MMP-8 processing of CXCL8 markedly activated the chemokine, with CXCL8 (6-77) leading to increased intracellular Ca^++^ mobilization (Figure 7A) and commensurate cell migration in transwells *in vitro* (Figure 7B). Despite several MMPs biochemically characterised to cleave and activate CXCL8, the critical importance of MMP-8 in CXCL8 activation was shown by injecting full-length CXCL8 in the air pouches of *Mmp8*-/- mice (Figure 7C). Here, the early PMN migration was decreased by >50% at 4 h compared with wild type mice. At later time points, there was less of a difference, but still it was always depressed in the knock out mice. *Mmp8*-/- PMN responsiveness and cell migratory behaviour *in vivo* was also shown to be unaffected by the absence of MMP-8 when challenged with the synthetic analogue of MMP-8-cleaved CXCL8 (6-77) (Figure 7C). This reconfirms the critical role of MMP-8 in directing chemotaxis by chemokine processing rather than cleavage of other molecules such as those in the blood vessel wall or extracellular matrix. Similar results were obtained using synthetic analogues of MMP-8-cleaved CXCL5 (8-78) compared with full-length CXCL5 (1-78) in the air pouch model (data not shown). In using human chemokines in a murine setting, it was important to show that rodent MMP-8 did cleave CXCL8 at the same site as human MMP-8 (Figure 6A, D). However, rodent MMP-8 cleaved human CXCL5 at Arg^9^∼Glu^10^ with no cleavage detected at Val^7^∼Leu^8^ (Figure 6D). Hence, these *in vitro* and *in vivo* studies indicate that similar to PMN migration mechanisms towards LIX in mice, human PMN chemoattraction in response to CXCL8 and CXCL5 also exhibits a unique MMP-8 dependent feed-forward activation mechanism.

## Discussion

We have addressed the role of MMPs in the activation of ELR^+^ CXC chemokines *in vivo*. Using *Mmp8*-/- mice, the essential role of PMN MMP-8 was shown in the activation pathway of the murine CXC chemokine LIX and human CXCL5 and CXCL8. Reflecting this, the absence of MMP-8 led to a profoundly defective PMN infiltration response *in vivo* to LPS or to full-length LIX, CXCL8, and CXCL5. This occurred despite the biochemical redundancy in chemokine activation by several commonly expressed MMPs including 1, 2, 9, 13, and 14 in a chemokine specific manner. PMN MMP-8 proteolysis leads to the activation of selected ELR^+^ CXC chemokines responsible for directing PMN cell migration and activation *in vivo*. With MMP-8 being primarily expressed by PMNs, our study identifies MMP-8 as an essential mediator of an interesting and unique activation mechanism of PMNs in innate immunity. This highlights an unexpectedly important role of the PMN itself in the integration of stimuli for the appropriate release of MMP-8 for LIX, CXCL8, and CXCL5 activation and so reveals an autologous cellular activation mechanism that acts in a feed-forward manner to orchestrate the PMN influx and LPS responsiveness.

Precise control of innate immunity is key for the development of successful inflammatory responses, resolution of infection, tissue healing, and ultimately host survival. As first line defence cells, the rapid recruitment and efficient activation of PMNs is of critical importance. It has long been known that the ELR^+^ CXCR2 cognate ligands that direct PMN chemotaxis and activation are expressed as relatively inactive ligands requiring proteolytic truncation of up to eight amino acid residues from the amino terminus for full activity, providing that the essential ELR motif remains intact [15]. However, relatively little attention has been focused on identifying the responsible enzymes *in vivo* and the location of chemokine activation—key questions for understanding the control of PMN function and innate immunity mechanisms. Naturally occurring NH_2_-terminal truncated forms of ELR^+^ CXC chemokines including LIX [17], [41], CXCL8 [42], CXCL1, 3 and 5 [43], have been reported but the proteases involved have not been identified *in vivo*. Mice deficient in MMP-9 [44], neutrophil elastase [45], both MMP-9 and neutrophil elastase [46], or cathepsin G [47] exhibit a normal PMN chemotactic response, indicating that PMNs neither require these enzymes for CXC chemokine activation, nor for migration and efficient chemotaxis *in vivo*. Hence, the deficient LPS and PMN migratory responses in the *Mmp8*-/- mouse are unique and reveal the singular importance of MMP-8 in these processes. However, we do not discount the possibility that *in vivo* other proteases downstream of MMP-8 activity may also activate LIX.

Consistent with previous observations [15], [34], [41] was our demonstration through the use of *in vitro* calcium flux and chemotaxis assays that the MMP-8-processed chemokine products LIX (5-92), LIX (5-79), CXCL8 (6-77), and CXCL5 (8-78) were more potent than the full-length chemokines. Notably, LIX (5-92) and (5-79) were equally potent chemokines indicating that the C-terminal cleavage at position Lys^79^∼Arg^80^ did not influence chemotactic activity. Chemokines interact with the highly negatively charged glycosaminoglycan chains of proteoglycans resulting in immobilization and the generation of a haptotactic gradient within the extracellular matrix that is responsible for directing leukocyte migration [48]. This has been confirmed physiologically for CC type chemokines [49] and murine ELR^+^ CXC chemokines [50]. Binding is typically through a heparin binding consensus sequence BBXB where B is a basic residue [51]. The MMP-8 C-terminal cleavage of LIX only removes two basic amino acids, both arginines, in the truncated product and so only slightly reduces heparin affinity. The biological significance of this cleavage is therefore unknown.

None of the MMPs analysed could cleave any of the other murine ELR^+^ chemokines (data not shown) revealing the specificity of MMP chemokine cleavage. This susceptibility of LIX to MMP processing is likely due to an extended N-terminus before the ELR motif, as compared with KC, MIP-2, and DCIP-1. Interestingly, rodent MMP-8 cleaved CXCL5 at Val^7^∼Leu^8^ and not at the same site as human MMP-8, Arg^9^∼Glu^10^. The active site of murine and rat MMP-8 differs from that of human MMP-8 by the presence of Lys^187^ at S_3_' instead of Ala^187^ as found in human MMP-8. Charge repulsion between Lys^187^ in rodent MMP-8 at S_3_' with the P_3_' Arg^12^ in CXCL5 may preclude CXCL5 cleavage at Arg^9^∼Glu^10^ and instead favour cleavage at Val^7^∼Leu^8^. Here Glu^10^ of CXCL5 would be the P_3_' residue and so likely forms a salt bridge with Lys^187^. Although LIX shows structural homology to CXCL5 and CXCL6 [52], MMP-8 exclusively cleaved CXCL5 and CXCL8 amongst all seven human ELR^+^ CXC chemokines. The activation of CXCL5 and CXCL8 were shown by *in vitro* calcium flux and chemotaxis assays, with the singular importance of MMP-8 in activating these human chemokines *in vivo* shown from studies comparing the *Mmp8*-/- mice with the wild type controls.

Our results reveal that chemokine processing is one of the most important functions of MMP-8 *in vivo* and casts into doubt the importance of MMP-8 in collagen degradation, a role that has long been assumed to be of particular importance for PMN cell migration and chemotaxis. Indeed, using air pouch models, no difference in PMN cell migration and infiltration was observed in response to synthetic analogues of the MMP-8 cleaved LIX, CXCL8, and CXCL5. This shows that the PMN cell migration machinery to chemotactic agents does not require MMP-8 activity for responsiveness and that MMP-8 proteolysis of blood vessel basement membrane and interstitial extracellular matrix components is not essential for effective cell migration *in vivo*. Although MMPs have traditionally been thought to cleave extracellular matrix components and so disrupt extracellular matrix contacts with the tumour and potentiate tumour cell spread and metastasis, other biological roles for MMPs in cancer are now known [20], [53]. Potentially related to this, sustained inflammatory responses maintain a microenvironment advantageous to tumour growth [54]. Indeed, MMP-8 modulates the innate immune response induced by carcinogens leading to a protective role in preventing tumour progression [34]. Mice lacking MMP-8 exhibited an abnormal inflammatory response upon application of carcinogen, with a delayed and more diffuse PMN influx to the site of the host challenge. Once established though, the inflammatory response was sustained and the mechanism for this is under investigation in our laboratory. With MMP-8 being the first MMP reported to have a protective role in tumorigenesis [34], the recognition of further MMP anti-targets in cancer continues [35]. MMP-3 has a protective role in squamous cell carcinoma [55] and macrophage MMP-12 is an anti-target in lung carcinoma [56]. In these cases the proteases were reported to alter leukocyte infiltration, although the mechanism and substrates were not elucidated. In view of the considerable number of chemokines now known to be processed by MMPs, chemokines are strong candidate substrates to phenotypically explain cancer anti-target activity in these MMP genetic knockout mice.

The role of MMPs in LPS responsiveness and PMN migration differs from that found for macrophages and CXCR4-displaying leukocytes. Instead of promoting cell migration, MMP cleavage of CC chemokines CCL2, -7, -8 and -13 results in the loss of agonist activity and the generation of potent *in vitro* and *in vivo* CCR antagonists [18], [19]. Interestingly, the MMP-2 cleavage and inactivation of CXCL12 [25] in the brain generates a potent and selective neurotoxin implicated in HIV dementia [57], [58]. We also recently found that MMP-2 induces the shedding of the integral plasma membrane chemokine CX3CL1 (fractalkine) by release of the chemokine domain from the stalk at Ala^71^∼Leu^72^ [23]. Further, the cell surface agonist activity of CX3CL1 was converted to a soluble antagonist due to processing at Gly^4^∼Met^5^. Hence, MMPs dynamically regulate the biological activity of chemokines and inflammatory and immune cell function in pleiotropic ways. Our present studies suggest that an *in cis* feed-forward activation mechanism occurs in which the PMN integrates the tissue signalling milieu leading to controlled release of MMP-8 that either directly or indirectly activates LIX in the mouse, and CXCL8 and CXCL5 in man for further PMN migration.

## Materials and Methods

### Animals

Mice deficient in *Mmp8* on a C57BL6/J×129 S background were provided by Dr. S. Shapiro (Boston, USA). Wild type C57BL6/J×129 S mice were purchased from the Jackson Laboratory. Animal breeding and experimental procedures were approved by the Animal Care Committee of the University of British Columbia. 6–8 week old mice, segregated according to sex, were used for all experiments.

### *In vivo* PMN chemotaxis

The air pouch model of PMN chemotaxis was used as described previously [59]. Sterile air (3 mL) was injected under the dorsal skin of mice, two days later the air pouch was reinflated with 2 mL of sterile air. On day five, 1 µg LPS (Sigma) in phosphate buffered saline or 5 µg of chemokine in 1 mL 0.5% carboxymethylcellulose was injected into the air pouch. After 0, 4, 8, or 12 h the mice were sacrificed and air pouches lavaged with 2 mL of sterile PBS. The resulting cell suspensions were lysed with 0.1% Triton-X100 and freeze-thawed. The PMN content was determined by myeloperoxidase activity [60] using isolated PMN cells as a standard. Cell lysate aliquots were separated by 7.5% SDS-PAGE and MMP-8 was detected by western blot with 1∶10,000 rabbit IgG against recombinant mouse MMP-8 [34].

### Chemokines and proteinases

LIX (1-92), LIX (5-92), LIX (5-79), MIP-2, KC, DCIP-1, CXCL8, CXCL8 (6-77), CXCL5 and CXCL5 (8-78) were chemically synthesized and purified [61]. Recombinant human MMP-1, -2, -8, -9, -13, -14, rodent MMP-8, -13, and recombinant human MMP-8 hemopexin C-domain were expressed and purified [18], [19]. The synthetic hydroxamate MMP inhibitor Batimastat (BB94) was from British Biotech (Oxford, UK).

### Chemokine cleavage assays

Analysis of chemokine cleavage by MMPs was performed at enzyme/chemokine ratios from 1∶1000 up to 1∶10 (w/w), at 37°C in assay buffer (150 mM NaCl, 20 mM Tris, 5 mM CaCl_2_, pH 7.5) in the presence of 1 mM APMA to activate proMMPs for 16–22 h. Digests were terminated by adding 5 µM EDTA. Recombinant MMP-8 hemopexin C-domain, EDTA or BB94 were added to cleavage assays as indicated. Reaction products were analyzed by 15% Tris-Tricine SDS-PAGE and stained with Coomassie Brilliant Blue R250. The [M+H]^+^/z of each cleavage product was determined by matrix assisted laser desorption ionization time of flight (MALDI-TOF) mass spectrometry on a Voyager-DE™ STR Biospectrometry Workstation (ABI). Mass spectrometry data was deconvoluted to identify the substrate cleavage sites and confirmed by Edman sequencing.

### Heparin binding

To assess the effect of MMP truncations of LIX on heparin binding, 0.5 mL of 4 µM chemokine in 10 mM potassium phosphate, pH 7.5 was loaded onto a 1 mL Hitrap^TM^ heparin-Sepharose column (GE Healthcare). Bound LIX and synthetic analogues of MMP-cleaved LIX were eluted using a linear gradient of 0 to 1.0 M NaCl over 20 min at a flow rate of 1.0 mL/min and monitored by in-line absorbance at 215 nm.

### Cells

Murine PMNs were isolated from bone marrow as previously described [46] except PMNs were recovered from a density gradient comprised of Histopaque 1077 layered on top of Histopaque 1119 according to manufacture's instructions (Sigma, St Louis, USA). The murine pre-B 300-19 cell line stably expressing human CXCR2 was supplied by Dr. B. Moser (Bern, Switzerland). Cells were cultured in RPMI-1640 medium containing 10% fetal bovine serum, 1% glutamine and 5×10^−5^ M β-mercaptoethanol (Sigma) under puromycin (1.5 µg/mL) selection.

### Measurement of intracellular calcium mobilization

Murine PMNs or CXCR2-transfected B300-19 cells (1×10^7^/mL in RPMI-1640 media supplemented with 1% serum) were incubated with 2 µM Fluo-4-acetoxymethyl ester (Molecular Probes), for 30 min, 37°C. Cells were washed to remove unincorporated agent and resuspended at 1×10^6^ cells/mL in Hanks Balanced Salt Solution (Gibco), 20 mM HEPES, 2.5 mM probenecid (Sigma). The cells were allowed to equilibrate at 37°C for 5 min prior to addition of ligand as indicated. Calcium concentration was monitored by excitation at 485 nm and emission of 520 nm with a PerkinElmer LS50B spectrophotometer. Calibration was performed by addition of 5 µM ionomycin (Sigma) and 1 mM MnCl_2 _(Fisher Biotech).

### *In vitro* chemotaxis assays

Murine PMNs or CXCR2-transfected murine pre-B cells were plated (1×10^6^/well in αMEM, 0.1% BSA) in transwell inserts (pre-coated with 10% fetal bovine serum) containing 3 µm or 8 µm pores respectively (Costar). Chemokines were added to the lower chamber and the plates incubated at 37°C. PMN chemotaxis assays were performed for 1 h. The migrated cells were then fixed with 4% paraformaldehyde prior to counting. The murine pre-B cell transfectants were assayed for 4 h after which the migrated cells were counted.
