# Correction: LPS responsiveness and neutrophil chemotaxis in vivo require PMN MMP-8 activity

**DOI:** 10.1371/journal.pone.0339233

**Published:** 2025-12-19

**Authors:** Angus M. Tester, Jennifer H. Cox, Andrea R. Connor, Amanda E. Starr, Richard A. Dean, Xose S. Puente, Carlos López-Otín, Christopher M. Overall

After publication of this article [1], concerns were raised regarding Fig 1. Specifically, there appears to be a vertical discontinuity between lanes 1 and 2 in Fig 1B.

The corresponding author stated that the WT and KO mouse cell lysate protein lanes were placed side by side in Fig 1B, but they were not side by side in the original gel (S1 File). They stated that two lanes from the same blot (S1 File), lane 4 (KO) and lane 7 (WT), were placed adjacent to each other in Fig 1B for ease of comparison. An updated version of Fig 1 is provided here, in which all four contiguous lanes of the WT and KO mice cell lysates blotted for MMP8 in the air pouch model are presented in Fig 1B as they were electrophoresed in the original gel (S1 File). The corresponding author also stated that Fig 1B is of *in vivo* fluid from an air pouch model, and therefore cytosolic loading controls were not blotted. Instead, equal amounts of protein from equivalent numbers of cells (corresponding to 50,000 cells) were loaded and the gels were analyzed on an equal-cell basis.

PLOS considers the above concerns regarding Fig 1B resolved.

The corresponding author also provided the available underlying data supporting other published results (S2–S12 Files), including replicate data for Fig 2A from the time of the original experiments (S9 File) and from earlier experiments (S8 File).

## Supporting information

S1 File Original uncropped image of the western blot from Fig 1B. (JPG)

S2 File Original gel scan in support of Fig 2A. (TIF)

S3 File Original gel scan in support of Fig 2C. (TIF)

S4 File Original gel scan in support of Fig 2D. (TIF)

S5 File High resolution version of Fig 6B. (TIF)

S6 File Original image in support of Fig 6A. (TIF)

S7 File Individual-level underlying quantitative data for Fig 1A. Cell count data and volumes of air pouch fluid and lysate for the air pouch experiments in Fig 1A, where 24 male mice were used, comprised of WT (*n* = 12) and KO (*n* = 12) mice treated with saline controls (*n* = 4) or LPS (*n* = 8) each.
(PDF)

S8 File Independent earlier experiments in support of Fig 2A with three of the mouse CXCL chemokines (LIX, KC and MIP-2). DCIP-1 was later synthesised and included in new assays for cleavage of the four mouse CXCL chemokines (S9 File).
(PDF)

S9 File Repeat TRIS-tricine gel analysis of independent assay samples of rat and human MMP8 digests performed on different days and from the time of the original experiments in support of Fig 2A. (ZIP)

S10 File Original gel image in support of Fig 3A. (TIF)

S11 File Underlying data for calcium flux assays, chemotaxis numbers, heparin binding, PMN incubations and receptor binding in Fig 5. (ZIP)

S12 File Raw AKTA data used to generate the heparin sepharose elution profile graph in Fig 2E. The heparin-Sepharose chromatography elution profile generated by a Pharmacia AKTA plotting the individual A280 absorption data points collected continuously during protein elution. This includes Excel files and original AKTA.pzf files for full-length LIX (LIX 1–92) and the synthetic cleaved analogues (LIX 5–79; LIX 5–92). The columns in LIX 1–92 are not labelled but are the same as labelled in the other Excel sheets for the cleaved forms: ml, A215, ml, % B.
(ZIP)

**Fig 1:**
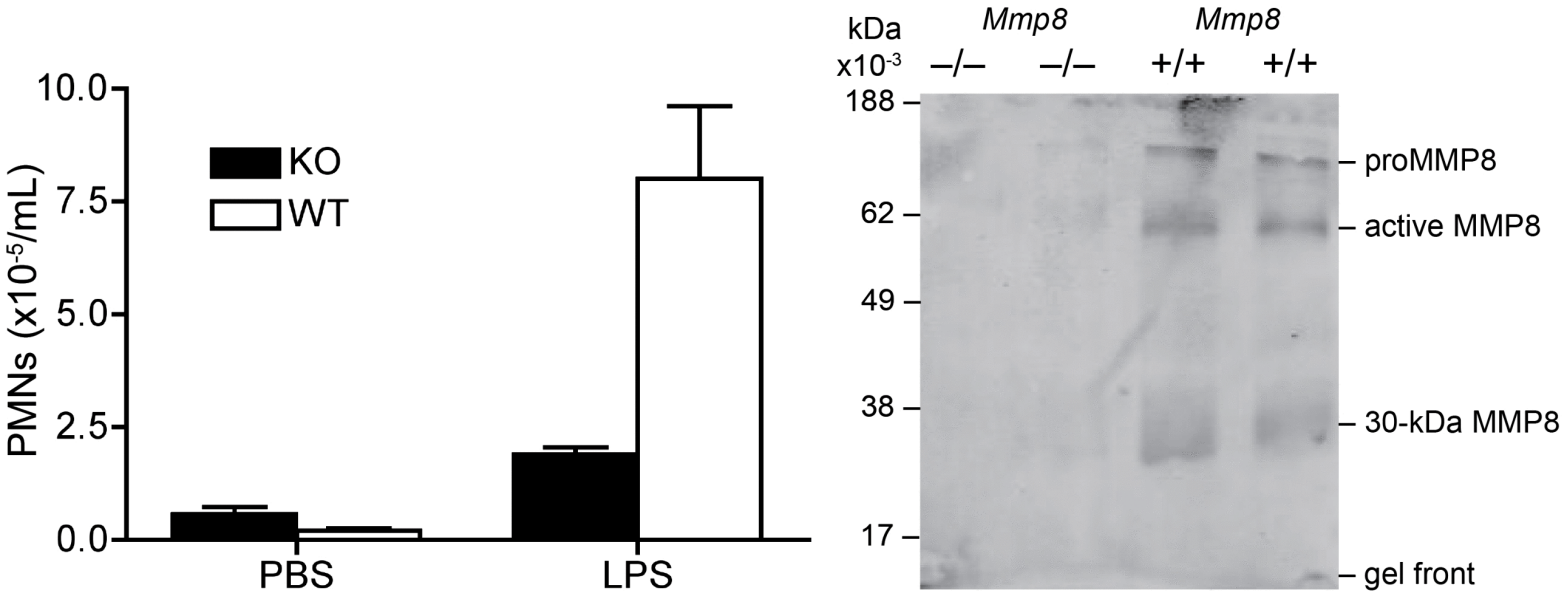
Impaired PMN responsiveness to LPS in *Mmp8*-null mice. (A) Infiltration of PMNs *in vivo* in response 1 mg of LPS (*n* = 8) or phosphate-buffered saline control (*n* = 4) injected into the air pouch of male *Mmp8* –/– (black bar) and wild-type (*Mmp8* + /+) male mice (white bar) was assessed 8 h post-injection. The PMN influx was quantified by myeloperoxidase activity. Error bars, standard error of the mean. (B) Western blot analysis of murine MMP-8 in the LPS-treated air-pouch PMN lysates corresponding to 50,000 cells per lane from *Mmp8* –/– (*n* = 2 of *n* = 8) and *Mmp8* + /+ (*n* = 2 of *n* = 4) mice. The figure panel was revised to display the four PMN lysates that had been electrophoresed on the same gel, but in the original Fig 1B, only lane 1 (knock out) and lane 4 (wild type) were displayed adjacent to each other without clear separation.
