# Supplementary figures and images for: Correction: LPS responsiveness and neutrophil chemotaxis in vivo require PMN MMP-8 activity
Source: PLoS One. 2025 Dec 19;20(12):e0339233. doi: 10.1371/journal.pone.0339233 (PMC12716691; doi:10.1371/journal.pone.0339233)

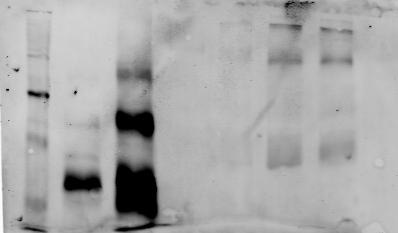

Supplement: S1 File — (JPG) [file pone.0339233.s001.jpg]

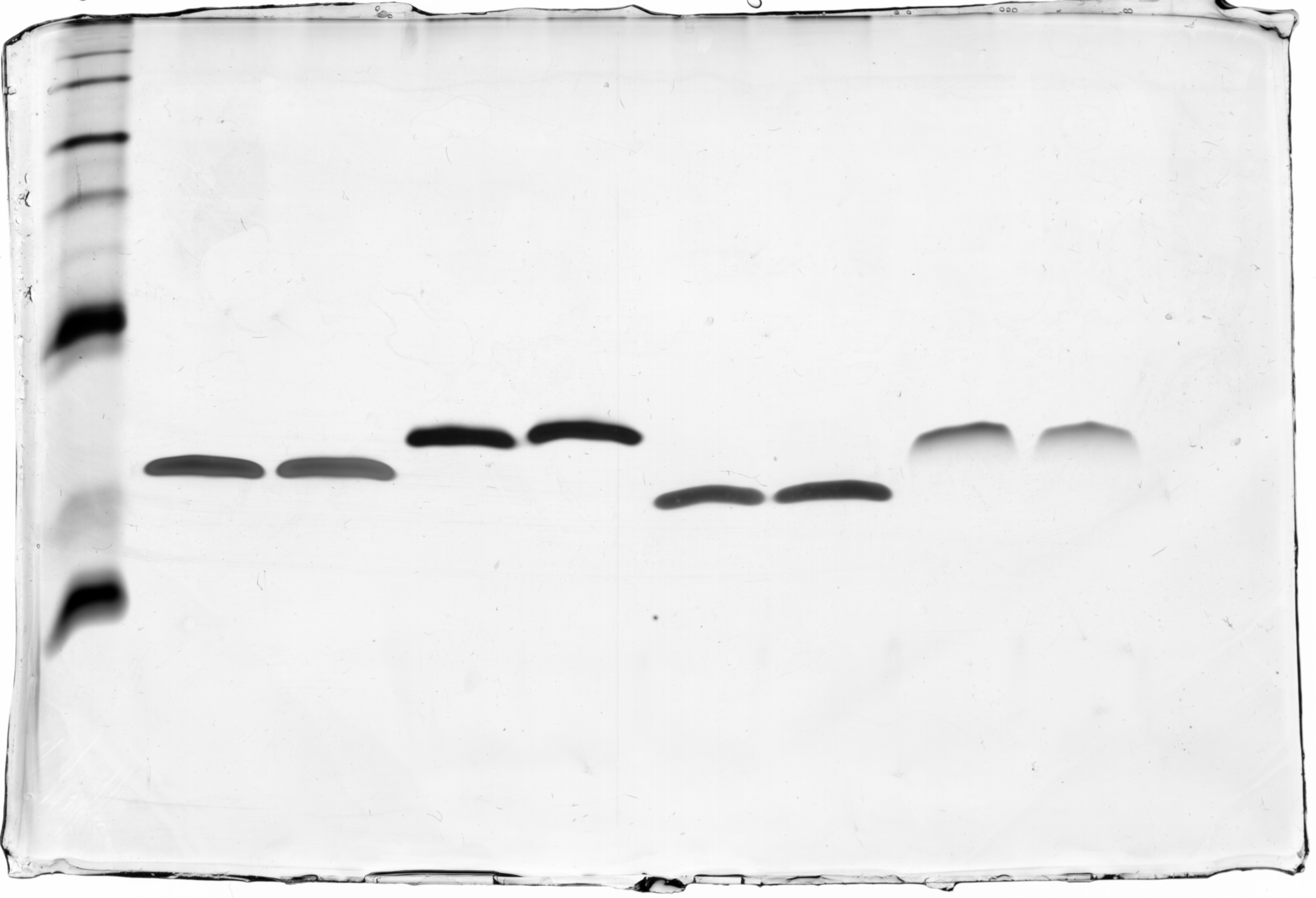

Supplement: S2 File — (TIF) [file pone.0339233.s002.tif]

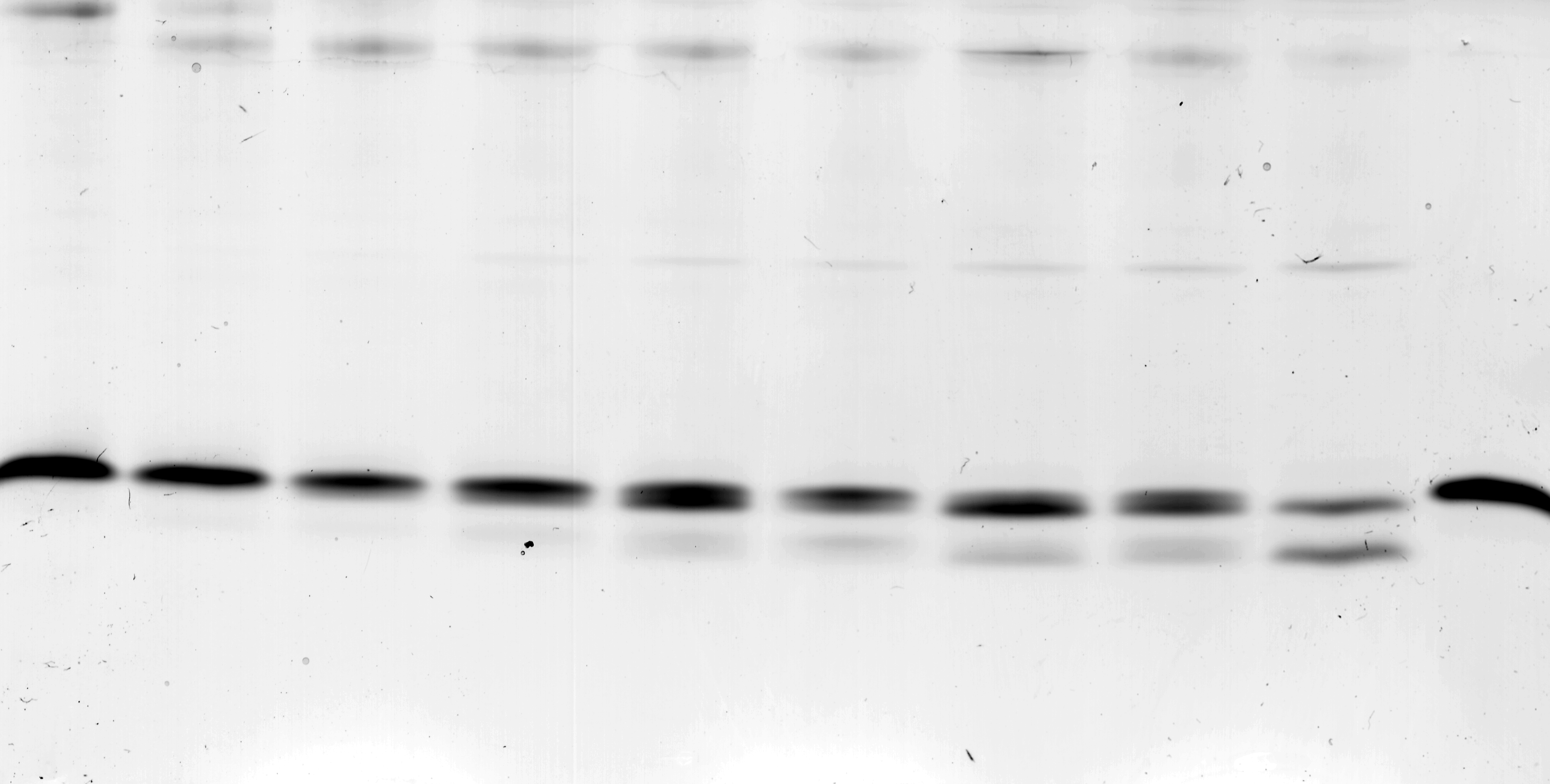

Supplement: S3 File — (TIF) [file pone.0339233.s003.tif]

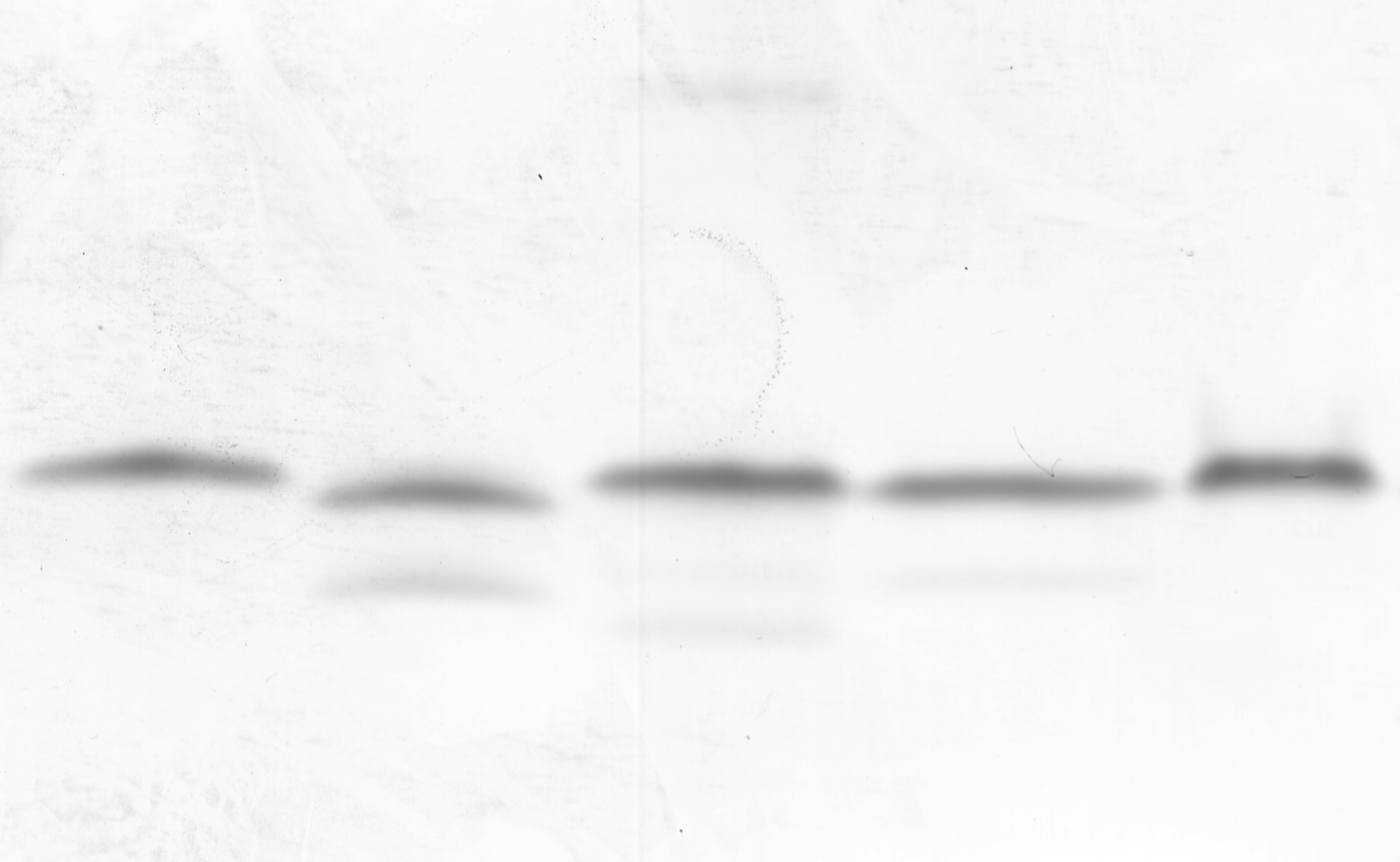

Supplement: S4 File — (TIF) [file pone.0339233.s004.tif]

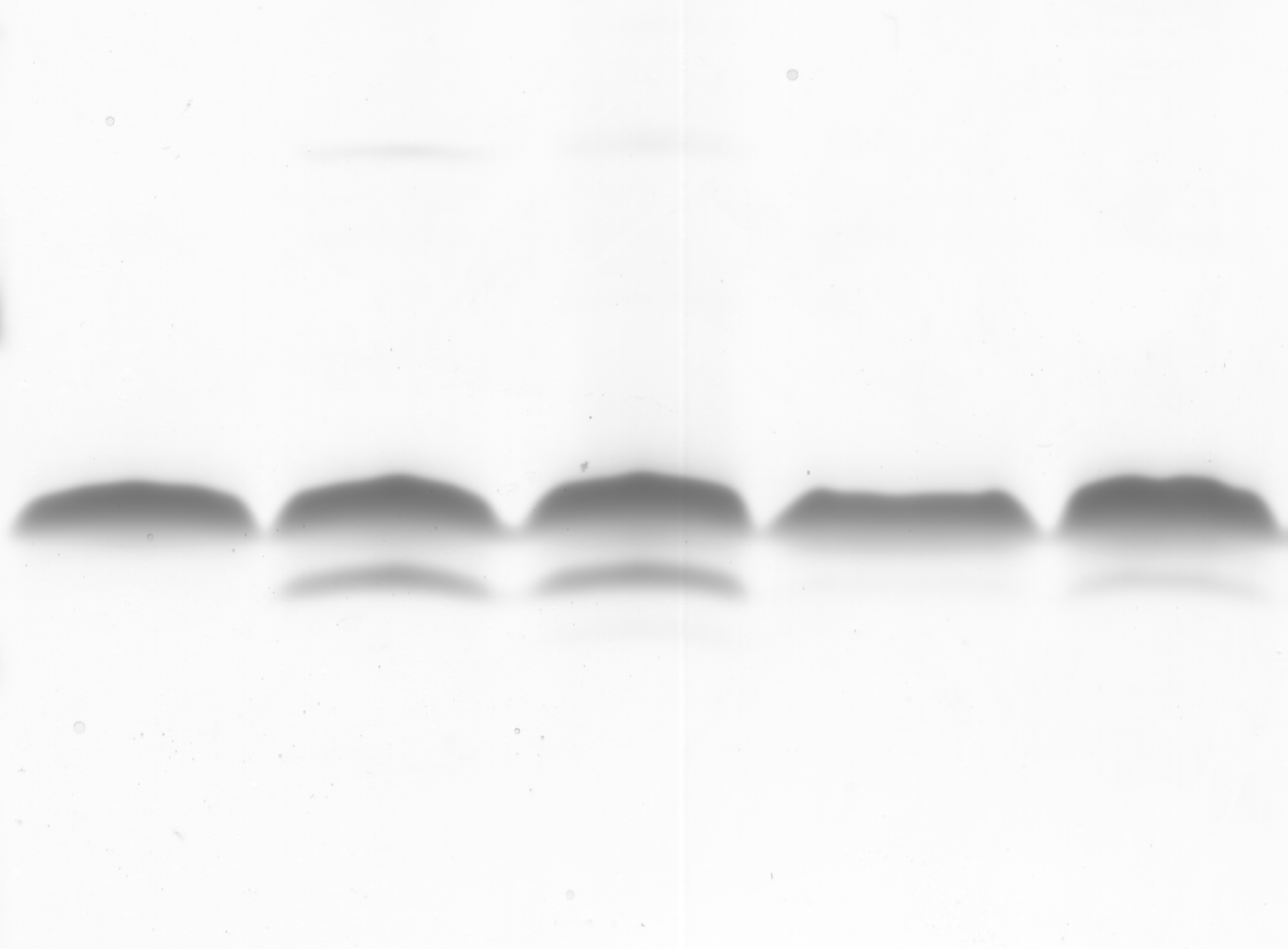

Supplement: S5 File — (TIF) [file pone.0339233.s005.tif]

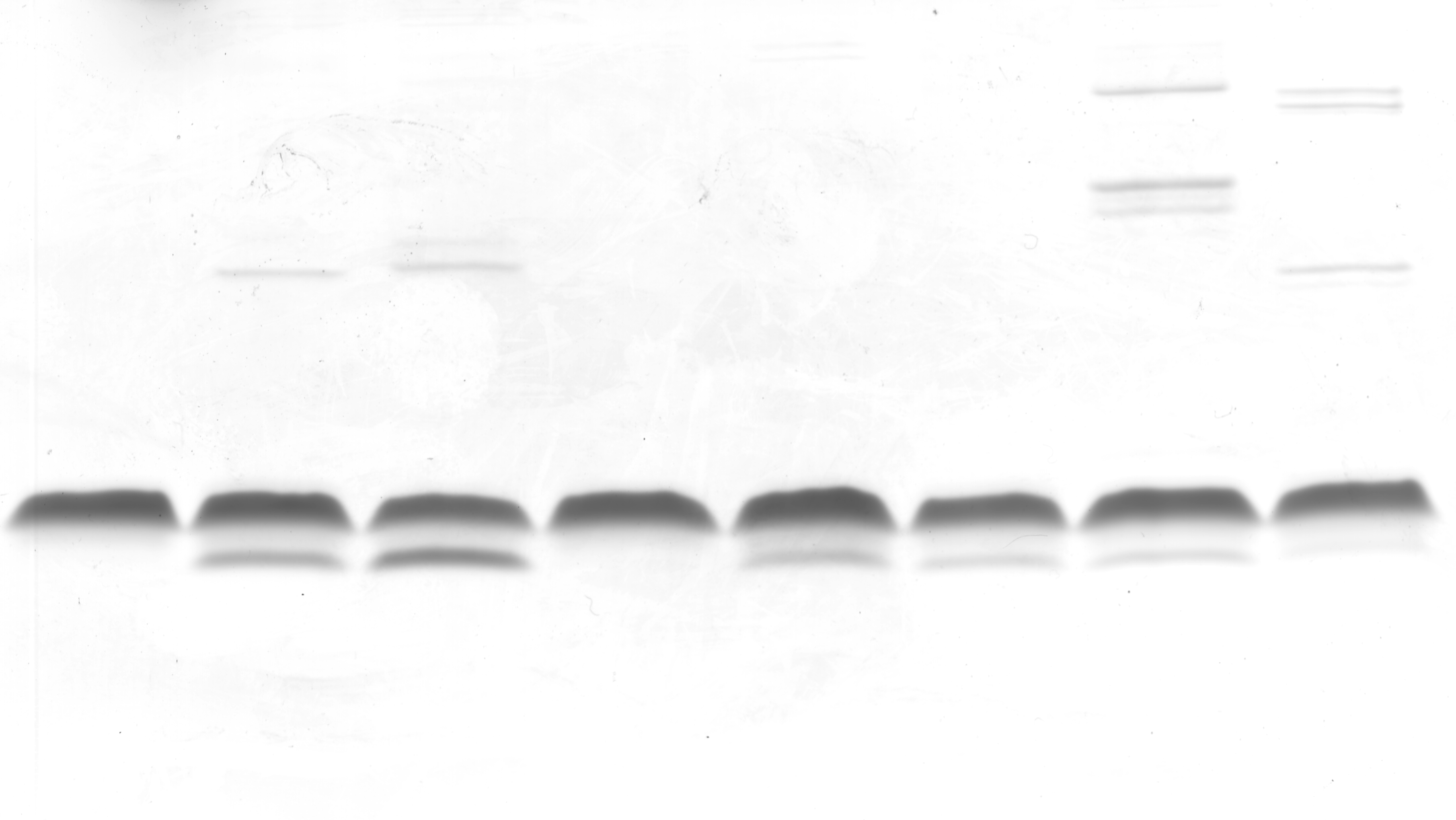

Supplement: S6 File — (TIF) [file pone.0339233.s006.tif]

Figure 2, Tester et al

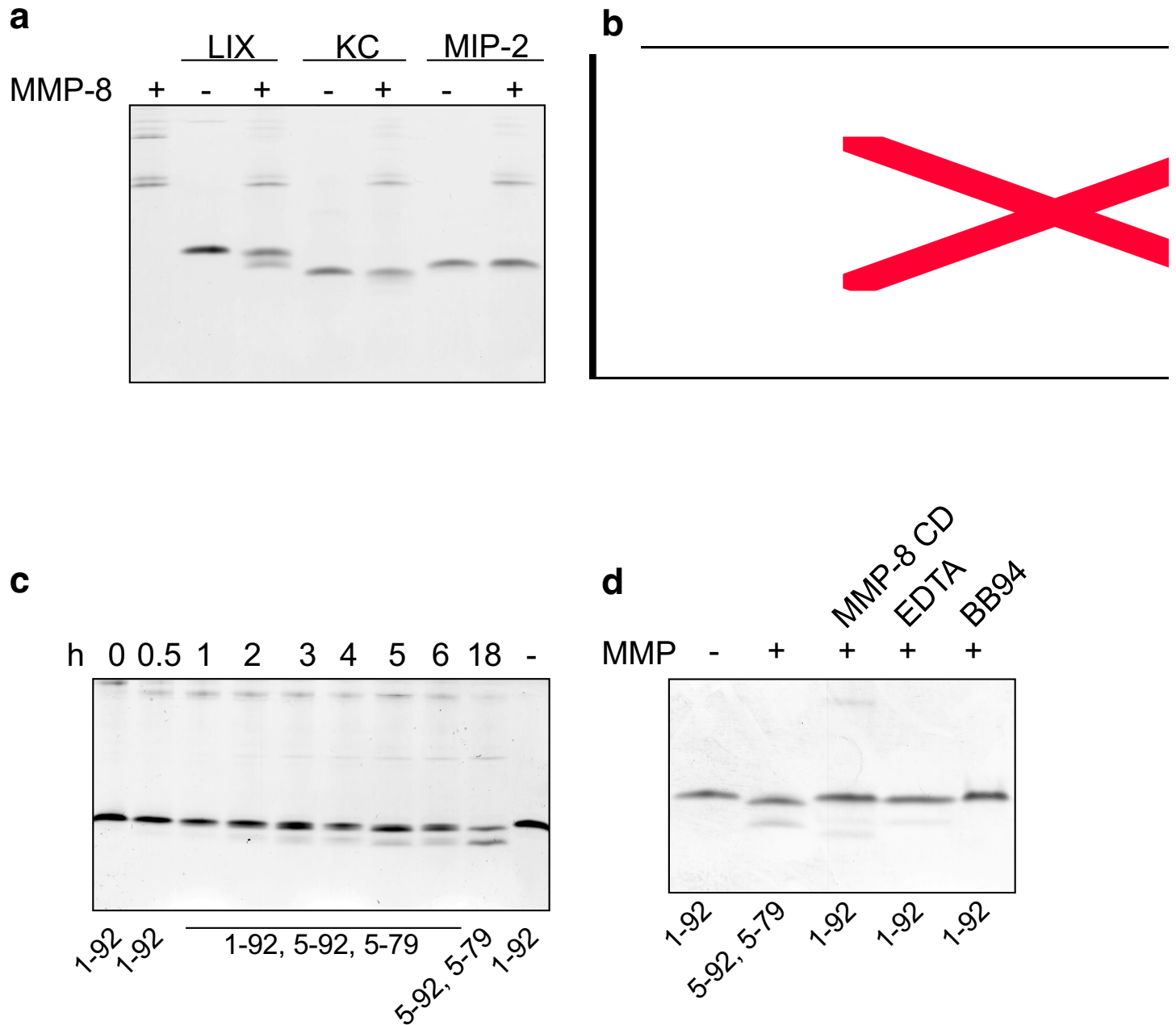

Supplement: S8 File — DCIP-1 was later synthesised and included in new assays for cleavage of the four mouse CXCL chemokines (S9 File). (PDF) [file pone.0339233.s008.pdf]

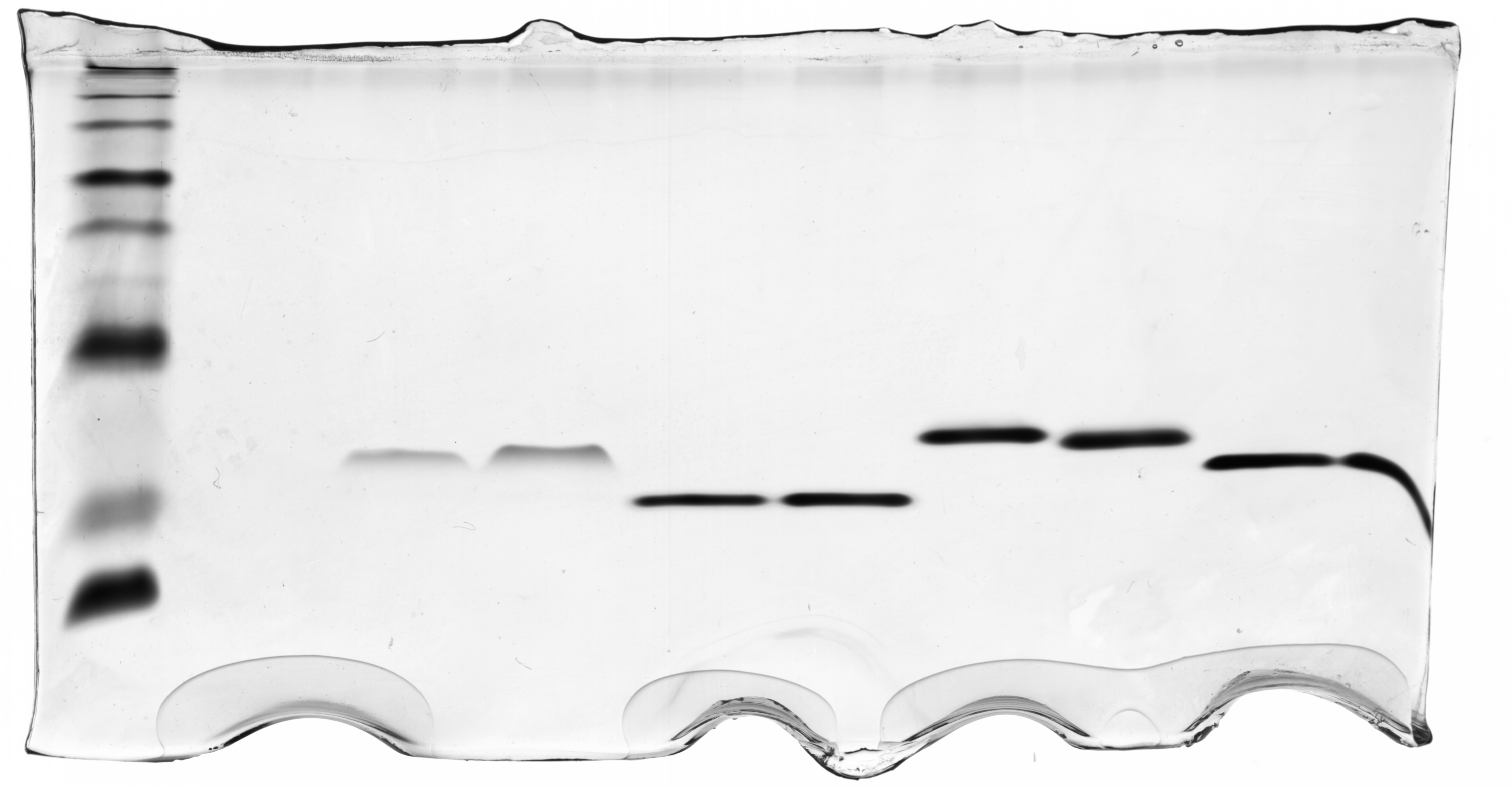

Supplement: S9 File — (ZIP) [file pone.0339233.s009.zip › 16aug0601b.tif]

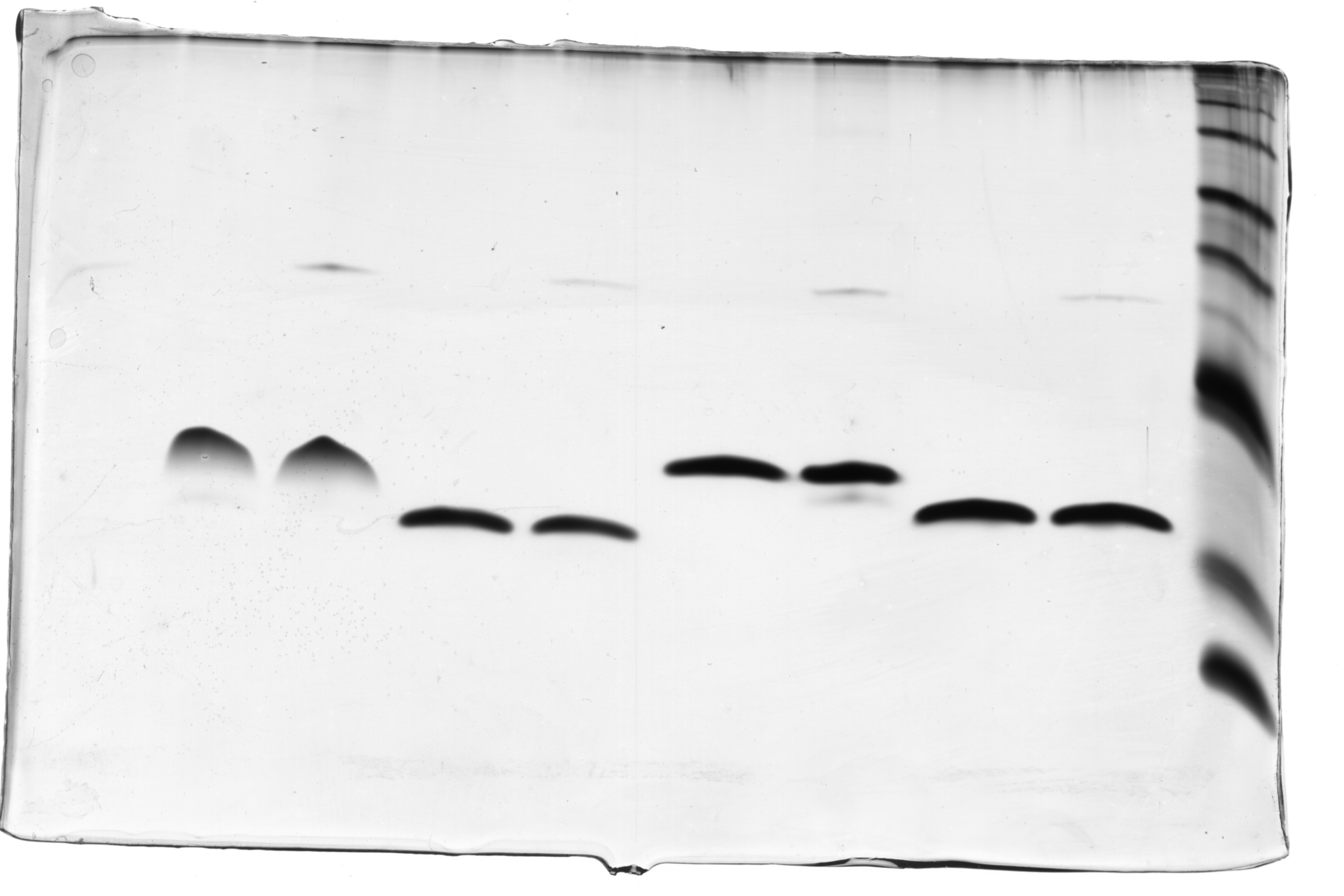

Supplement: S9 File — (ZIP) [file pone.0339233.s009.zip › 18aug0601a human.tif]

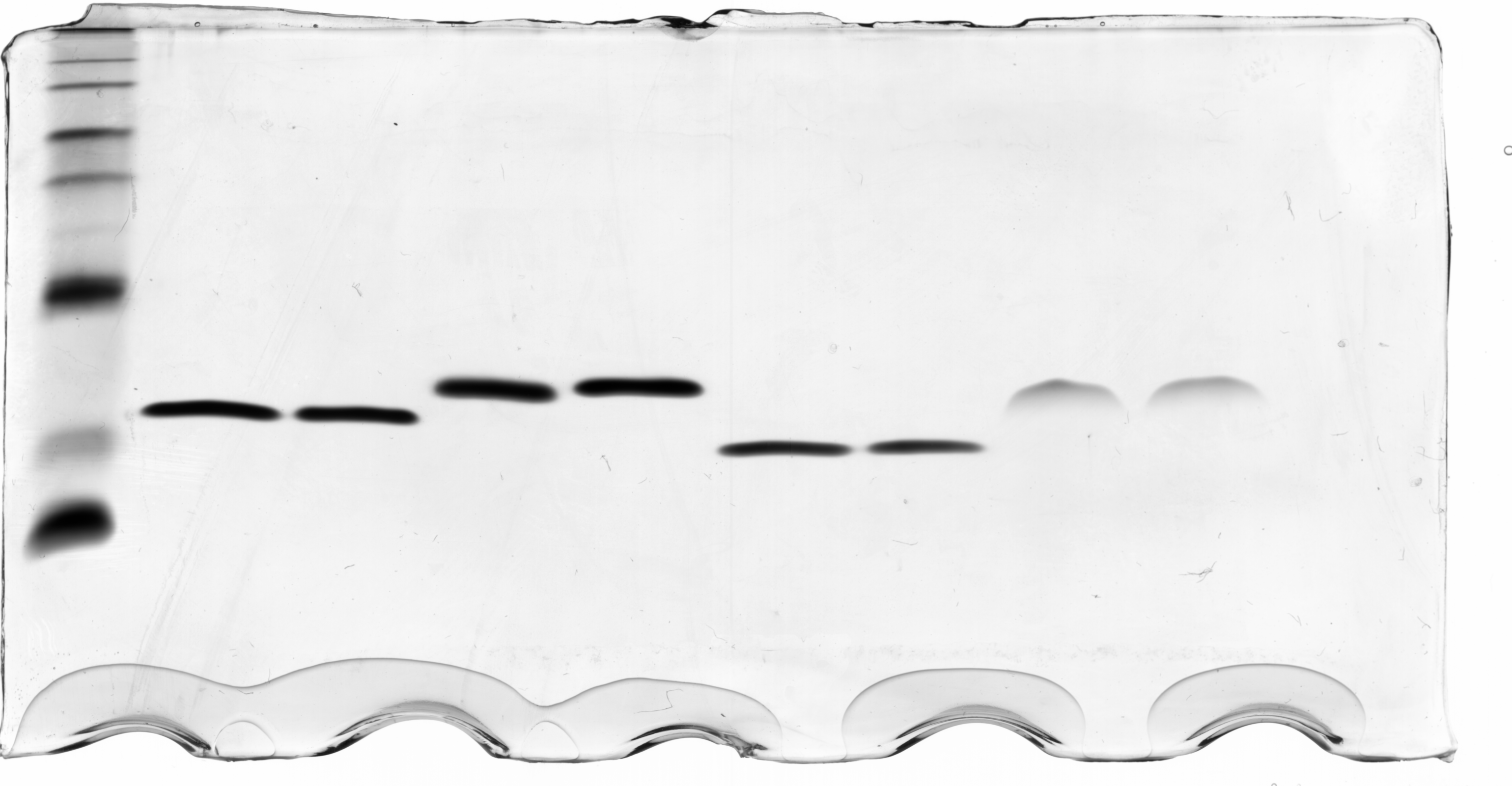

Supplement: S9 File — (ZIP) [file pone.0339233.s009.zip › 22aug0601b.tif]

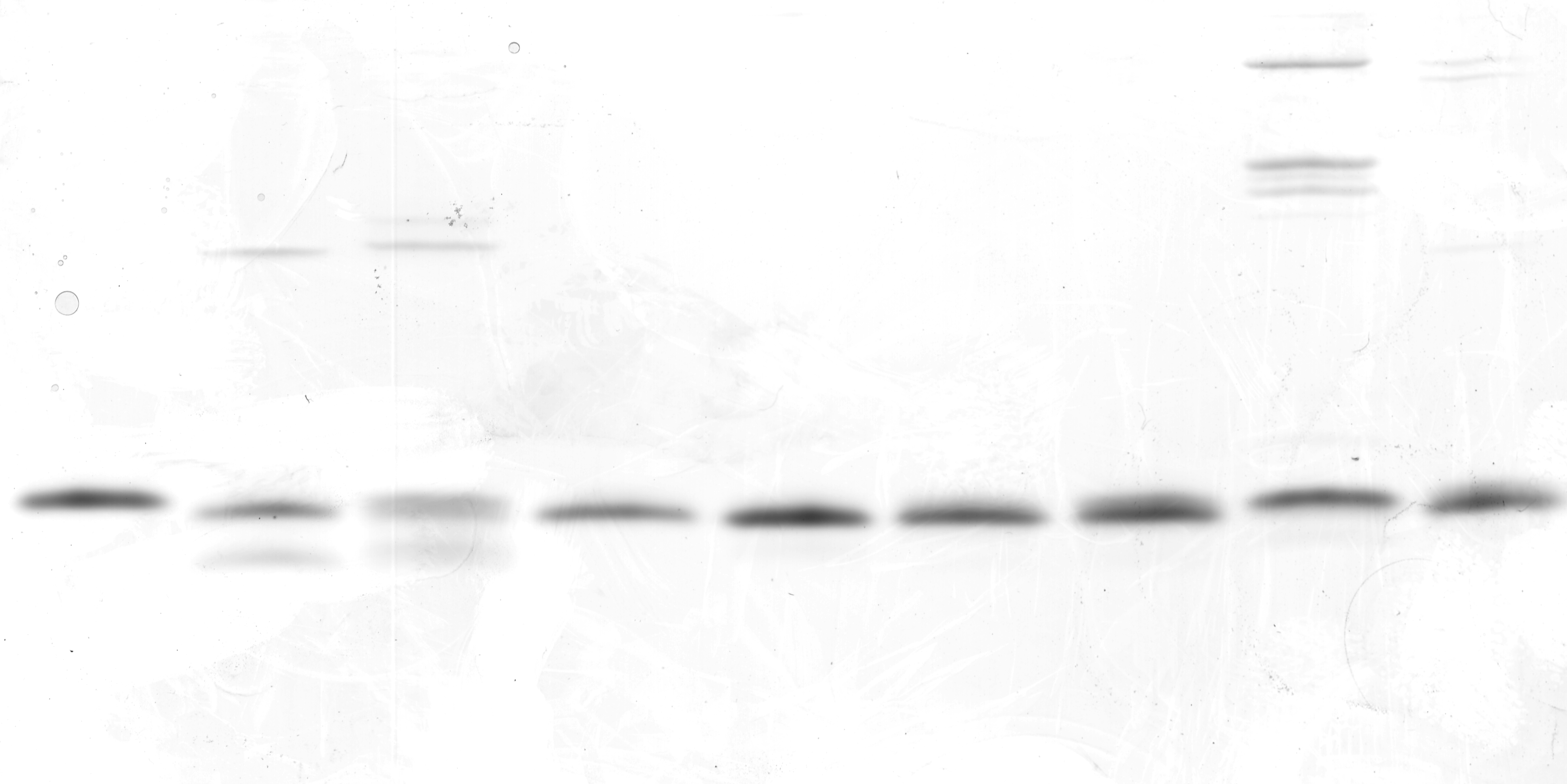

Supplement: S10 File — (TIF) [file pone.0339233.s010.tif]
