# Supplementary material for: Correction: LPS responsiveness and neutrophil chemotaxis in vivo require PMN MMP-8 activity
Source: PLoS One. 2025 Dec 19;20(12):e0339233. doi: 10.1371/journal.pone.0339233 (PMC12716691; doi:10.1371/journal.pone.0339233)
Supplement: S11 File — (ZIP) [file pone.0339233.s011.zip › 06422635 Fig 5 Calcium Flux/16jun04fluxfigs.doc]

16jun0405: 100 nM

16jun0406: 50 nM

16jun0407: 10 nM
